# Gratitude in Organizations: Psychometric Properties of the Italian Version of the Gratitude Resentment and Appreciation Test–Revised Short (GRAT–RS) in Workers

**DOI:** 10.3390/ijerph191711084

**Published:** 2022-09-04

**Authors:** Letizia Palazzeschi, Andrea Svicher, Alessio Gori, Annamaria Di Fabio

**Affiliations:** 1 Department of Education, Languages, Intercultures, Literatures and Psychology (Psychology Section), University of Florence, 50135 Florence, Italy; 2 Department of Health Sciences (Psychology Section), University of Florence, 50135 Florence, Italy

**Keywords:** gratitude, organizations, workers, psychometric properties, Gratitude Resentment and Appreciation Test–Revised Short (GRAT–RS), healthy organizations, strength-based prevention perspectives

## Abstract

In a healthy organization’s framework and strength-based prevention perspectives, gratitude represents an important resource. Gratitude is a worthy construct able to promote the well-being of both workers and organizations. Gratitude is also an interesting variable in relation to success, efficiency and productivity in organizations that can be increased through specific training. Gratitude is, therefore, considered a promising resource for both individuals and organizations. This study aims at analyzing the psychometric properties of the Italian version of the Gratitude Resentment and Appreciation Test–Revised Short (GRAT–RS). Participants were 359 Italian workers from different public and private organizations. Factor structure, internal consistency, and concurrent validity were analyzed. Confirmatory factor analysis supported a multidimensional bifactor structure. Good internal consistency and concurrent validity were established. Results indicated that the Italian version of the Gratitude Resentment and Appreciation Test–Revised Short (GRAT–RS) is a valid instrument to detect gratitude in the Italian context with workers.

## 1. Introduction

In the psychological realm, there is an increased interest in the study of gratitude [1,2,3]. There are several reasons why gratitude could be an interesting and promising construct to explore [4], in particular, in the organizational field. First, research has highlighted that gratitude is essential to individuals and being grateful is considered not only to be an aspect of particular value [5] but above all as an important resource for the person [6] promoting individual well-being [7]. 

The term gratitude comes from the Latin gratia which refers to thankfulness or grace [6]. Gratitude can be felt by human beings and non-human entities. It derives from a cognitive process including two phases: (1) individuals acknowledge that they have obtained a favorable outcome related to happiness feelings; (2) individuals ascribe their happiness feelings to sources different from the self, creating a connection between happiness and gratitude [8]. Furthermore, gratitude could be defined as an empathic emotion [9] because individuals experience gratitude only when they acknowledge and value what another person did for them [10]. McCullough et al. [5,7] consider gratitude as an individual disposition in terms of a widened tendency to identify and reply with grateful emotion to others’ benevolence, especially when individuals are moving toward one’s favorable outcomes and experiences. Thus, the literature also emphasizes the importance of emotional aspects in gratitude, referring to a grateful affect and considering gratitude as an affective characteristic [4].

### 1.1. Theoretical Structure of Gratitude

The theoretical structure of gratitude is an open issue [11]. Some researchers suggest that gratitude is a unitary concept [7], represented as a unidimensional model in latent factor theory. Others advanced the idea that gratitude is not unitary [4,12,13,14], reflecting distinct but correlated dimensions. In the factor realm, it is described through a correlated common factors model [15]. Following these considerations, Watkins et al. [4] merged these positions conceiving a comprehensive model of gratitude. It is composed of three different dimensions, namely the pillars of gratitude (sense of abundance; appreciation of simple pleasure; appreciation of others) that exist simultaneously and are also linked with a superordinate dimension of gratitude [11]. According to the factor theory, the superordinate dimension of gratitude can be modeled as a general factor existing independently of pillars (bifactor model) or running through them (second order model) [15]. Furthermore, the literature also illustrates different views on the theoretical contents of the factorial models [16,17,18]. This discussion encompasses the question of whether appreciation (often regarded as a subset of gratitude) is separate from the superordinate dimension of gratitude [16,17], encompassing also an ongoing debate on whether appreciation is or is not separate from the concept of gratitude [16,17,18]. More broadly, there is no unitary consensus on which specific forms of gratitude have to be distinct to add incremental utility over the general concept of gratitude [11]. For example, differently from the three pillars of gratitude, Morgan et al. [19] developed a multi-dimensional measure of gratitude, the Multi-Component Gratitude Measure (MCGM) to detect four dimensions of gratitude: conceptions of gratitude, grateful emotions, attitudes toward gratitude (including motivational aspects and judgment of its relevance), and gratitude-related behaviors. However, the instrument is not a self-reported questionnaire but participants are presented with scenarios to detect their understanding of gratitude. Lastly, intersected with the abovementioned theoretical issues, there are questions on the reliability of self-report measures of gratitude [11]. In this framework, the Gratitude Resentment and Appreciation Test–Revised Short form (GRAT–RS) [13] emerged as a widely used psychometrically sound measure.

### 1.2. Construct Dimensionality of GRAT-RS

The 16-item GRAT-RS was developed by Thomas and Watkins [13] by reducing the original three factors of the 44-item Gratitude Resentment and Appreciation Test (GRAT) [14]. According to the GRAT, grateful people are considered to have the following characteristics: firstly, they should not perceive feelings of deprivation regarding their lives and they should feel a sense of abundance; secondly, they should have a tendency to appreciate simple pleasures; finally, they should recognize the role of other people in their well-being [2,4]. Watkins et al. [2,4] took these characteristics of individuals into account to create the items of their questionnaire to detect gratitude. The questionnaire GRAT permits thus to individuate three dimensions of gratitude: (1) Lack of a sense of deprivation (sense of abundance) in relation to one’s own life; (2) Simple appreciation regarding simple pleasure; (3) Appreciation of others in terms of appreciation of the role of others in one’s own life [4]. Afterward, Froh et al. [20] further examined the factor structure of GRAT-RS via both exploratory factor analysis (EFA) and confirmatory factor analysis (CFA). After removing one item, they confirmed a three-factor orthogonal solution [20]. In the same study, the authors supported the 15-item version of the GRAT–RS in a sample of young people aged from 10 to 19 [20]. More recently, Hammer and Brenner revised the factor structure of GRAT–RS by comparing all the theoretical structures of gratitude operationalized via four factor models: unidimensional model, three-factor orthogonal model, second order model, and bifactor model [11]. 

### 1.3. Gratitude in Organizational Contexts

In the psychological domain, gratitude was traditionally studied in the realm of positive psychology [21], especially in relation to well-being [4,7,22,23]. More recently, research has also concentrated on working environments [1,2], expanding research from the association between gratitude and well-being [24] to the associations with other constructs, particularly relational aspects, such as positive interpersonal interactions and social support at work [25]; prosocial organizational behaviors [26,27], organizational citizenship behaviors, teamwork and altruism [28]. Gratitude is also promising for job performance, efficiency, and productivity [24,29], thus permitting the promotion of better performances in healthy organizations [30,31,32,33]. According to these premises, the positive organizational approach has introduced the concept of collective gratitude in the working context, defining it as a positive emotional disposition (being thankful for the favorable events that occur) shared within the working group [34]. Subsequently, Fehr et al. [35] proposed a model of gratitude in organizations according to a multilevel perspective, addressing the event, individual, and organizational levels. Fehr et al.’s [35] multilevel model comprises episodic gratitude at the event level, persistent gratitude at the individual level, and collective gratitude at the organizational level. Episodic gratitude regards an occurrence that arouses emotional feedback of appreciation that is beneficial to individuals but not ascribable to the self [35]. Persistent gratitude is a long-lasting disposition to feel grateful within certain circumstances [35]. Collective gratitude concerns enduring gratitude expressed by components of an organization [35]. However, Fehr et al. [35] did not develop specific instruments to detect gratitude at these different levels.

In strength-based prevention perspectives [36] with a specific focus on a primary preventive level [37,38,39,40,41], gratitude is considered a strength for individuals and it is amenable to specific training [42,43].

The current world of work characterized by new technologies, digitization, automation, and globalization is also perceived as unstable and unsure [44,45]. To respond to the new challenges of the 21st century new resources are required [46,47,48,49,50] and gratitude could be such a promising resource at every level promoting virtuous circles in organizations. For example, it could also support managers [51] who cope with the current scenario to promote people as healthier workers [52].

In organizational contexts, the use of brief scales is particularly useful because it permits to reduce the times of administration and the costs of research for organizations but allows for the preservation of high reliability of the measurement according to accountability principles [53,54], and this could be useful also in relation to instruments to detect gratitude.

An attempt to develop a scale to measure gratitude for organizations was made by Wnuk [55] who realized the Gratitude Toward the Organization Scale in the Polish context. This questionnaire is composed of 12 items and has two subscales: gratitude as a commitment to reciprocity and gratitude as a moral norm. 

### 1.4. Purpose of the Current Study

According to the previously mentioned framework, it seems thus significant to have a brief scale, internationally recognized, to measure gratitude in workers even in the Italian context. Therefore, the aim of this study is to analyze the psychometric properties of the Italian version of the Gratitude Resentment and Appreciation Test–Revised Short (GRAT–RS) [4,11,13] as a traditional brief measure of gratitude, internationally recognized, and with the characteristic of brevity particularly useful in organizational contexts.

## 2. Method

### 2.1. Participants and Procedure

Three hundred fifty-nine workers of private and public organizations in central Italy participated in the present study, 166 males (46.24%) and 193 females (53.76%) with a mean age of 37.77 (SD = 10.68). Participants were employees of different private and public organizations in the commercial, educational and health sectors. Participants were recruited via organizational gatekeepers. Participation was voluntary. Participants provided written and informed consent according to privacy Italian laws (Law Decree DL−196/2003) and European Union General Data Protection Regulation (EU 2016/679). The order of administration was counterbalanced to control the effects of the order of presentation. Participants with missing data were excluded. The final number of participants included in the analysis (n = 359) was judged adequate in line with the literature [56,57], highlighting that factor analysis validity is acceptable with more than 300 participants. 

### 2.2. Measures

#### 2.2.1. Gratitude Resentment and Appreciation Test–Revised Short (GRAT–RS)

The Italian version of the GRAT–RS [11,13] is made up of 16 items with response formats from 1 = Strongly disagree to 5 = Strongly agree. The items of the English version have been translated using the back translation method. The original version of GRAT-RS [11] showed a bifactor structure enclosing a general factor and three specific factors (lack of a sense of deprivation [LOSD], simple pleasures [SP], and social appreciation [SA]). The coefficient omega for the original GRAT-RS ranges from 0.92 to 0.85 [11]. An example of an item for the LOSD factor is “Life has been good to me”; for SP factor is “Oftentimes I have been overwhelmed at the beauty of nature” and for SA factor is “I couldn’t have gotten where I am today without the help of many people” [11].

#### 2.2.2. Positive and Negative Affect Schedule (PANAS)

The PANAS ([58]; Italian version [59]) is formed of 20 adjectives (10 for Positive Affect PA and 10 for Negative Affect NA). The respondents were asked to rate the extent to which they usually feel on average on a five-point Likert scale (1 = Very slightly or not at all; 5 = Extremely). Positive Affect (PA) measures the extent to which people feel active enthusiastic and determined. Negative Affect (NA) refers to subjective distress and adverse emotional states [58]. The Italian version showed psychometric properties in line with the original one, confirming a two-factor solution and showing Cronbach’s alpha between 0.90 (PA) and 0.84 (NA) [59]. An example of an item for PA is “interested”. An example of item for NA is “distressed” [58].

#### 2.2.3. Satisfaction with Life Scale (SWLS) 

The SWLS ([60]; Italian version [61]) is composed of five items on a Likert scale ranging from 1 = Strongly disagree to 7 = Strongly agree. It is designed to assess a global dimension of life satisfaction. The Italian version confirmed the unidimensional structure of the English SLWS, showing a Cronbach’s alpha of 0.88 [61]. An example of an item is “In most ways my life is close to my ideal.” [60].

#### 2.2.4. Flourishing Scale (FS)

The FS ([62]; Italian version [63]) encompasses eight rated response options ranging from 1 (Strongly disagree) to 7 (Strongly agree). The FS measures human flourishing in relevant domains, such as optimism, relationships, and purpose in life. The Italian version was found consistent with the original ones, displaying a one-factor structure with a Cronbach’s alpha of 0.88 [63]. An example of an item is “I lead a purposeful and meaningful life.” [62].

### 2.3. Data Analysis

The factorial structure of the Italian version of the GRAT–RS was verified by means of a Confirmatory Factor Analysis (CFA) through AMOS with the maximum likelihood method. Four models were examined: unidimensional (all items load on a single general factor), correlational (three oblique correlated factors), second order (three factors are regressed onto a second order factor), and bifactor (items are simultaneously regressed on their respective three factors and onto a general factor). Models were analyzed considering different fit indices: the Comparative Fit Index (CFI) [64] and the Non-Normed Fit Index (NNFI) [65] (values greater than 0.90 show a good fit; [64]); the Root Mean Square Error of Approximation (RMSEA) and the Standardized Root Mean Square Residual (SRMR) [66] (values lower than 0.08 show good fit; [67]). The reliability of the Italian version of the GRAT–RS was analyzed by calculating the Cronbach alphas. Furthermore, to verify some aspects of concurrent validity, the correlations of the Italian version of the GRAT–RS with measures of hedonic well-being (PANAS and SWLS) as for the study of the original version, and in addition with a measure of eudaimonic well-being (FS) were examined, using the Pearson coefficient *r*.

## 3. Results

To verify the bifactor model of the Italian version of the GRAT–RS, a series of confirmatory factor analyses were carried out. Table 1 shows the Goodness of Fit indices concerning the four tested models (unidimensional, correlational, second order, and bifactor).

**Figure 1:**
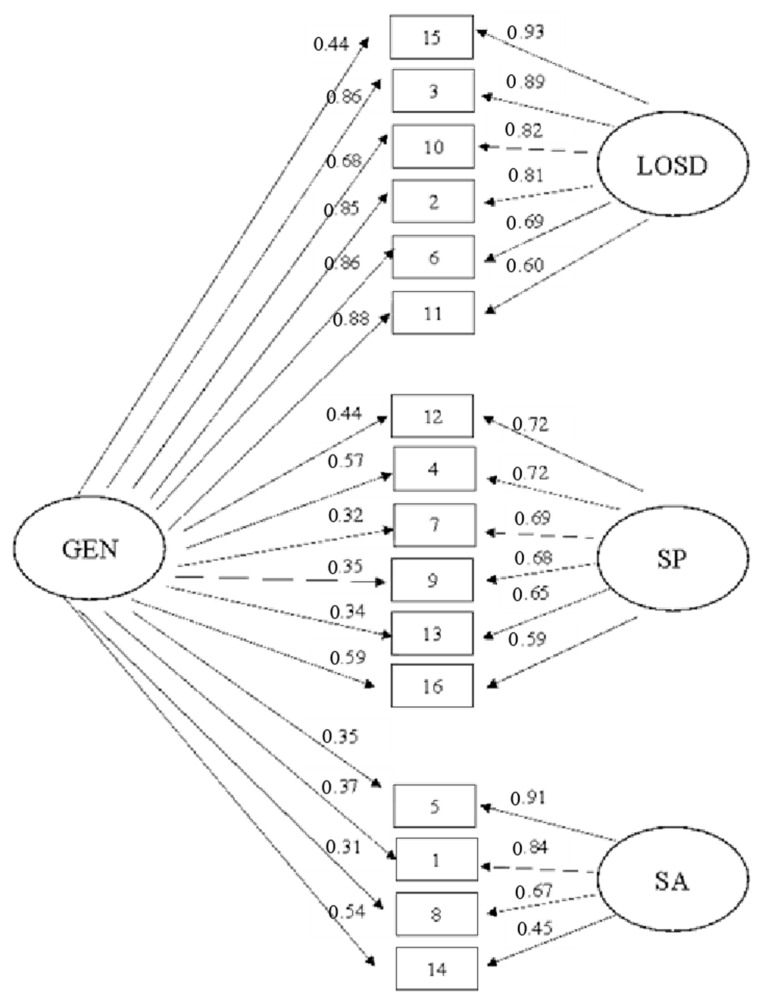
Bifactor measurement model of Italian version of the GRAT–RS (GEN: general factor; LOSD: lack of a sense of deprivation specific factor; SP: simple pleasures specific factor; SA: social appreciation specific factor).

Concerning the indices considered, the Italian version showed the best fit for the multidimensional bifactor model that demonstrated an acceptable fit across all indexes. Differently, the unidimensional, correlational, and second order models showed an unacceptable fit. Figure 1 also showed the standardized loadings for the bi-factor model. 

In order to verify the internal consistency of the questionnaire, Cronbach’s alphas were calculated. Following the bifactor measurement model, a Cronbach alpha was computed for the overall scale (total score) reflecting the general factor, and three Cronbach alphas were estimated for the three individual factors. (see Table 2).

## 4. Discussion

The purpose of this work was to analyze the psychometric properties of the Italian version of the GRAT–RS. An advantage of the GRAT–RS beyond other tools to measure gratitude is its possibility to offer a more comprehensive assessment with its three dimensions and total score [13]. The factor structure of the Italian version of GRAT-SF was investigated through CFA. Compared with unidimensional, correlational and second order models, the bifactor model showed the best fit. This indicates as suggested by Hammer and Brenner [11] that the covariation among the sixteen items of GRAT–RS could be explained by the occurrence of (1) a superordinate general gratitude factor, reflecting the overall items’ common variance; (2) three specific factors that reflect the additional and unique variance obtained by clustering the items into three dimensions [68].

The reliability of the three dimensions that emerged and the total score of the scale is adequate. The correlations of the Italian version of the GRAT–RS with the PANAS and the SWLS as measures of hedonic well-being and with the FS as measures of eudaimonic well-being underlined an adequate concurrent validity regarding the measurements carried out. Specifically, gratitude was associated both with affective aspects of hedonic well-being (directly with positive affect and inversely with negative affect) and with cognitive related to life satisfaction in a positive direction. It is also positively associated with aspects connected to the flourishing of individuals in terms of success perceived by themselves in crucial life domains, including relationships, self-esteem, purpose, and optimism [62]. These findings highlighted how gratitude is linked to well-being as it emerges in literature [4,6,17,23].

From the results of this study, it is possible to conclude that the Italian version of the GRAT–RS is a trustworthy and reliable instrument for assessing gratitude in the Italian context with workers. However, this study has the limitation of having analyzed the psychometric properties of the Italian version of the GRAT–RS only with workers of central Italy who are, therefore, not representative of the Italian reality. Forthcoming studies could thus use workers more representative of the Italian reality, enclosing participants from other geographic areas in Italy. It would also be interesting to study in-depth the psychometric properties of the GRAT–RS assessing its criterion validity via an SEM model [11], adding additional testing of divergent validity, as well as using alternative frameworks with respect to the classical test theories, for example, Item Response Theory (IRT).

## 5. Conclusions

Even considering these limitations, the Italian version of the GRAT–RS represents an instrument able to detect in accurate manner gratitude in the Italian context with workers. Having this instrument available could allow the opening of new research and intervention perspectives centering on gratitude as a new promising variable in strength-based prevention perspectives [36]; promoting the well-being of individuals and organizations and implementing a framework focused on healthy organizations [30,31] and decent work [69].

## Figures and Tables

**Table 1:** Confirmatory Factor Analysis of Italian version of the GRAT–RS: *Goodness of Fit*.

Models	RMSEA	SRMR	NNFI	CFI
**Unidimensional Model**	0.20	0.19	0.45	0.44
**Correlational Model**	0.11	0.08	0.83	0.86
**Second Order Model**	0.11	0.08	0.83	0.86
**Bi-factor Model**	0.07	0.06	0.91	0.92

RMSEA = Root Mean Square Error of Approximation; SRMS = Standardized Root Mean Square Residual; NNFI = Non-Normed Fit Index; CFI = Comparative Fit Index.

**Table 2:** Correlations of the Italian version of GRAT–RS with PANAS, SWLS and FS; Cronbach’s Alphas for the three dimensions and the total score of the Italian version of GRAT–RS.

	Cronbach’s Alphas	PA	NA	SWLS	FS
Lack of sense of deprivation	0.91	0.35 **	−0.30 **	0.30 **	0.33 **
Simple pleasures	0.83	0.31 **	−0.33 **	0.40 **	0.34 **
Social appreciation	0.83	0.30 **	−0.30 **	0.34 **	0.32 **
GRAT–RS total score	0.88	0.38 **	−0.31 **	0.44 **	0.45 **

*Note. N* = 359. ** *p* < 0.01. PA = Positive Affect; NA = Negative Affect; SWLS; = Satisfaction With Life Scale; FS = Flourishing Scale.

## Data Availability

The data presented in this study are available from the corresponding author on reasonable request. The data are not publicly available due to privacy reasons.
